# Global biomarker trends in triple-negative breast cancer research: a bibliometric analysis

**DOI:** 10.1097/JS9.0000000000001799

**Published:** 2024-06-10

**Authors:** Xingxin Wang, Xuhao Li, Tiantian Dong, Wenyan Yu, Zhixia Jia, Yi Hou, Jiguo Yang, Yuanxiang Liu

**Affiliations:** a College of Acupuncture-Moxibustion and Tuina, Shandong University of Traditional Chinese Medicine, Jinan, China; b Traditional Chinese Medicine External Treatment Center, Affiliated Hospital of Shandong University of Traditional Chinese Medicine, Jinan, China; c The First Clinical Medical College, Shandong University of Traditional Chinese Medicine, Jinan, China; d Department of Neurology, Affiliated Hospital of Shandong University of Traditional Chinese Medicine, Jinan, China

**Keywords:** Bibliometric, biomarkers, future trend, research topic, TNBC

## Abstract

**Background:**

Triple-negative breast cancer (TNBC) is defined as breast cancer that is negative for estrogen receptor (ER), progesterone receptor (PR), and human epidermal growth factor receptor-2 (HER-2) in cancer tissue. The lack of specific biomarkers makes the diagnosis and prognosis of TNBC challenging.

**Method:**

A comprehensive literature review and bibliometric analysis was performed using CiteSpace, VOSviewer and Scimago Graphica.

**Results:**

TNBC biomarker research has been growing rapidly in recent years, reflecting the enormous academic interest in TNBC biomarker research. A total of 127 journals published relevant studies and 1749 authors were involved in the field, with developed countries such as the United States, France, and the United Kingdom contributing greatly to the field. Collaborative network analysis found that the research in this field has not yet formed good communication and interaction, and the partnership should be strengthened in the future in order to promote the in-depth development of TNBC biomarker research. A comprehensive analysis of keywords and co-cited literature, etc. found that TNBC biomarker research mainly focuses on immune checkpoint markers, microenvironment-related markers, circulating tumor DNA, metabolic markers, genomics markers and so on. These research hotspots will help to better understand the molecular characteristics and biological processes of TNBC, and provide more accurate biomarkers for its diagnosis, treatment and prognosis.

**Conclusions:**

The bibliometric analysis highlighted global trends and key directions in TNBC biomarker research. Future developments in TNBC biomarker research are likely to be in the direction of multi-omics integration, meticulous study of the microenvironment, targeted therapeutic biomarkers, application of liquid biopsy, application of machine learning and artificial intelligence, and individualized therapeutic strategies. Young scholars should learn and collaborate across disciplines, pay attention to new technologies and methods, improve their data analysis skills, and continue to follow up on the latest research trends in order to meet the challenges and opportunities in the field of TNBC biomarkers.

## Introduction

Highlights

- High citation research: The study, referencing 217 articles with a total of 5553 citations, indicates that the field of triple-negative breast cancer (TNBC) biomarkers is an active and significant topic in medical research, receiving substantial attention from the academic community and clinical practice.
- Interdisciplinary academic focus: The research underscores the potential and importance of molecular biology and genetics in TNBC biomarker studies, revealing an interdisciplinary collaboration trend within this field of research.
- Future research directions: The study identifies current hotspots of research such as immune checkpoint biomarkers, tumor microenvironment, and circulating tumor DNA and proposes future directions including multi-omics integration and applications of machine learning, offering new perspectives and strategies for precise diagnosis and treatment of TNBC.

Triple-negative breast cancer (TNBC) is a subtype of breast cancer characterized by the absence of estrogen receptors, progesterone receptors, and human epidermal growth factor receptor 2 expression^1,2^. Statistical reports indicate that breast cancer has become the most prevalent malignant tumor among women, accounting for ~11.7% of cancer patients^3^. Compared to other subtypes, TNBC exhibits higher invasiveness, increased recurrence rate, and shorter overall survival^4,5^. Due to the absence of common hormone receptors and human epidermal growth factor receptor-2 (HER-2) expression in TNBC, conventional diagnostic methods often struggle with accurate identification. Consequently, researchers have been persistently seeking reliable biomarkers to aid in the diagnosis and prognostic assessment of TNBC.

Currently, researchers have achieved numerous significant advancements in the field of TNBC biomarker research. However, obtaining a comprehensive understanding of the overall progress and research trends in TNBC biomarker research poses challenges. Bibliometrics is a systematic research method used to assess and analyze publication patterns, academic impact, as well as trends and quality of research output within relevant fields^6^. This study employs bibliometric tools such as CiteSpace, VOSviewer, and Scimago Graphica to extract and transform implicit information from literature, thereby identifying influential authors, journals, institutions, and countries in this field. By generating collaboration network maps and temporal evolution diagrams, this study analyzes collaborative relationships and research hotspots in the TNBC biomarker field, revealing current challenges and underexplored areas. Additionally, we propose potential future directions for the development and utilization of biomarkers in clinical practice to advance TNBC treatment and management.

## Materials and methods

### Data sources and search strategies

**Figure 1:**
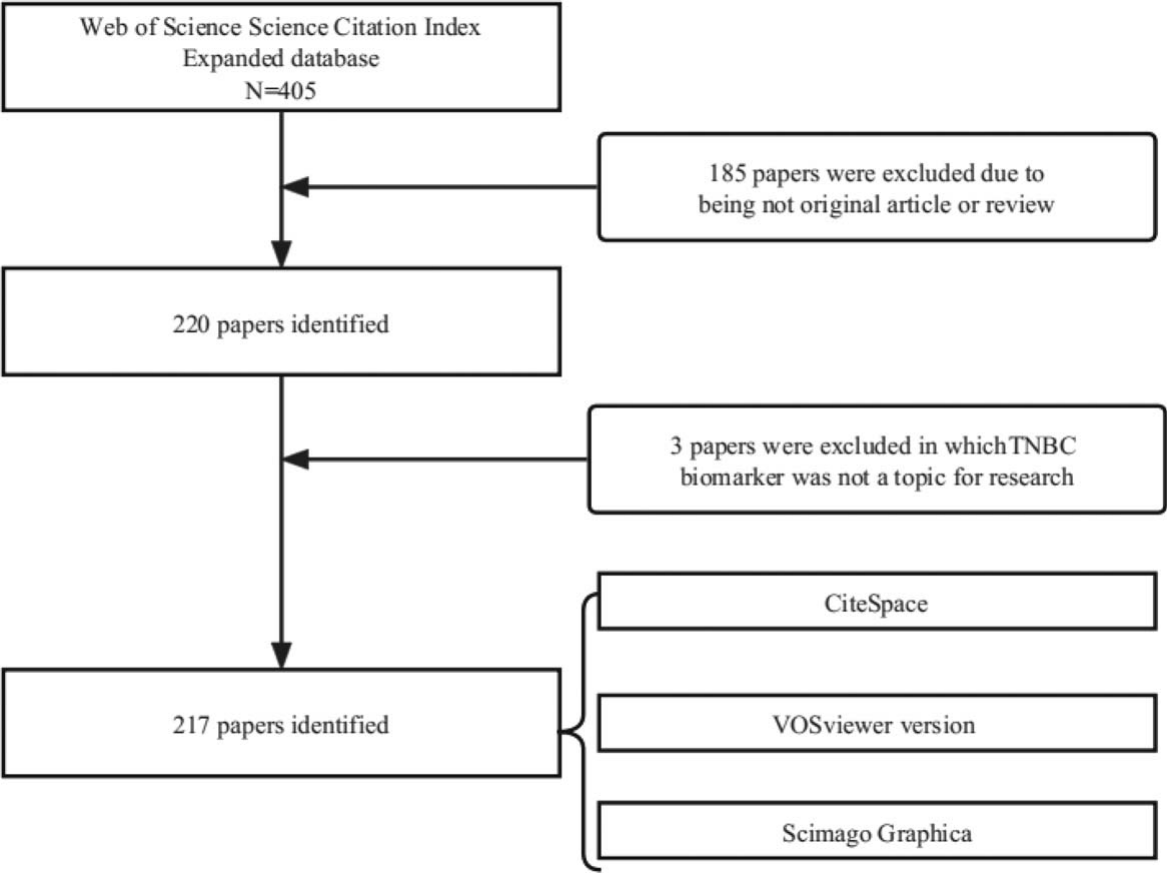
Flowchart of TNBC biomarker literature screening. TNBC, triple-negative breast cancer.

We utilized the largest and internationally recognized comprehensive academic information repository, Web of Science (WOS) Core Collection database, as our literature source. The following was the framework for the literature search: （TI=(ER-Negative PR-Negative HER2-Negative Breast Neoplasms) OR TI=(ER Negative PR Negative HER2 Negative Breast Neoplasms) OR TI=(Triple-Negative Breast Cancer) OR TI=(Breast Cancer, Triple-Negative) OR TI=(Breast Cancers, Triple-Negative) OR TI=(Triple-Negative Breast Cancers) OR TI=(Triple-Negative Breast Neoplasm) OR TI=(Breast Neoplasm, Triple-Negative) OR TI=(Breast Neoplasms, Triple-Negative) OR TI=(Triple Negative Breast Neoplasm) OR TI=(Triple-Negative Breast Neoplasms) OR TI=(ER-Negative PR-Negative HER2-Negative Breast Cancer) OR TI=(ER Negative PR Negative HER2 Negative Breast Cancer) OR TI=(Triple Negative Breast Cancer) )AND (TI= (biomarker*)). Relevant literature retrieved by JZX and LJL was imported into Zotero software for deduplication. Simultaneously, manual screening of titles and abstracts was conducted to exclude literature not pertinent to TNBC biomarker research. Any uncertainty regarding inclusion was resolved by Y.J.G. and L.Y.X. The search period for this study spanned from the inception of WOS up to 15 August 2023. Only articles and review articles published in English were considered. The quality of TNBC biomarker literature was re-evaluated by DTT, resulting in the final inclusion of 217 relevant studies (Fig. 1). W.X.X. exported TNBC biomarker literature in Refworks and Excel formats. Collaboration analysis among authors, institutions, and countries was conducted using VOSviewer and Scimago Graphica, while keyword and co-citation analysis were performed using CiteSpace software. By transforming the potential knowledge information from literature on TNBC biomarkers into a panoramic view, we aim to provide researchers in the field of TNBC biomarkers with a clear and comprehensive perspective, helping them better understand the current research status, anticipate future development directions, and foster scientific collaboration on a global scale.

## Trends related to TNBC biomarkers

### Articles

This study included a total of 217 literature on TNBC biomarkers, with a total citation of 5553. The paper titled “Long-term Clinical Outcomes and Biomarker Analyses of Atezolizumab Therapy for Patients With Metastatic Triple-Negative Breast Cancer A Phase 1 Study” by Emens, Leisha A. had the highest citation count (474 citations, IF=28.4, Q1, 2019)^3^. Following that is the paper titled “A randomised phase II study investigating durvalumab in addition to an anthracycline taxane-based neoadjuvant therapy in early triple-negative breast cancer: clinical results and biomarker analysis of GeparNuevo study” (357 citations, IF=50.5, Q1, 2019)^7^, “TBCRC009: A Multicenter Phase II Clinical Trial of Platinum Monotherapy With Biomarker Assessment in Metastatic Triple-Negative Breast Cancer” (314 citations, IF=45.3, Q1, 2015)^8^, “Identification and use of biomarkers in treatment strategies for triple-negative breast cancer subtypes” (299 citations, IF=7.3, Q1, 2014)^9^. The aforementioned articles have been cited over 200 times each. These highly cited papers all emphasize the role of biomarkers in predicting treatment responses and personalized medicine. Whether by assessing PD-L1 expression, BRCA1/2 gene mutation status, or exploring p63/p73 gene expression, the use of biomarkers is critical for optimizing treatment decisions. Moreover, they are published in top-tier journals with high-impact factors and widely cited, highlighting that biomarker research is currently a hotspot in the field of TNBC. This also reflects the latest developments in the field and the focus of the scientific community.

To better understand the activity level of literature in this field, we selected articles with a high “180 Day Usage Count” and analyzed them in conjunction with “Since 2013 Usage Count,” “Times Cited, All Databases,” “Publication Year,” and other factors^3,7,10–20^. By comparing literature from different years, we found that the focus of TNBC biomarker research is shifting from basic biomarker identification to understanding the specific mechanisms of these markers in cancer development. Secondly, the difference between high usage frequency and low citation counts may indicate that although these articles have received considerable attention recently, their academic impact has not been widely recognized. This phenomenon may be related to the novelty of the research or a sudden increase in research interest in specific fields. Furthermore, researchers are exploring new biomarkers (e.g. LncRNA T376626), which can be used not only for disease diagnosis and prognosis assessment but also as novel targets for therapy. Additionally, some researchers are focused on evaluating the effectiveness of specific treatment methods in TNBC therapy, emphasizing the application of biomarkers in clinical trials (e.g. durvalumab and atezolizumab). These 13 articles not only demonstrate the current status and trends of TNBC biomarker research but also reflect the in-depth exploration in the scientific research field regarding the discovery of new biomarkers, understanding disease mechanisms, and clinical applications. Through in-depth analysis of these articles, new researchers can identify research questions and design experiments in cutting-edge fields such as immunotherapy biomarkers, immune cell infiltration assessment, molecular classification and genomics, gene expression regulation biomarkers, radiomics, and mechanobiology, thus better planning their research development path.

### Year of publication and citation

**Figure 2:**
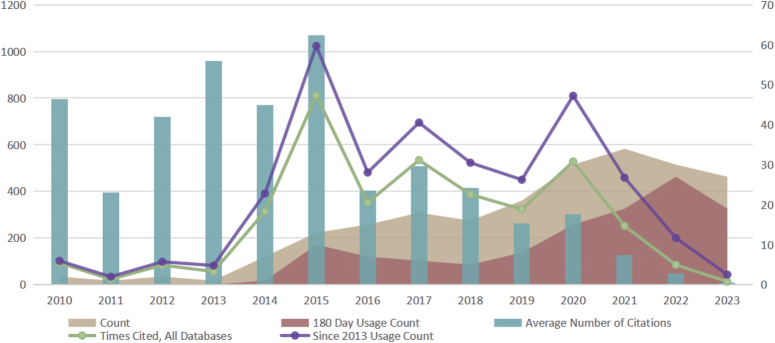
Year of publication and citations of triple-negative breast cancer biomarker literature.

To better understand the trends in TNBC biomarker development, we plotted a graph of the annual publication volume, total citation frequency, average citation frequency, and 180-day usage count in this field (Fig. 2). In terms of publication volume, TNBC biomarker literature has shown a significant upward trend year by year, with publication volume remaining consistently high in recent years, reflecting increasing research interest and activity among scholars in this field over time. Regarding citation frequency changes, although the overall publication volume is increasing, the average citation frequency of literature from each year fluctuates. For instance, although the publication volume was highest in 2021, its average citation frequency is relatively low (7 times), which may suggest that the literature from that year needs more time to demonstrate its academic value. Furthermore, literature from 2015 had the highest citation frequency (N=812), indicating the potentially high academic impact and importance of research conducted in that year. Analyzing the 180-Day Usage Count, there has been an increase in usage since 2018. Despite a decrease in publication volume during these two years, the short-term usage remains at a relatively high level, indicating significant attention to recent research findings and suggesting widespread interest among scholars in this field. Analysis of “Times Cited, All Databases” and “Since 2013 Usage Count” reveals that literature from certain years (e.g. 2015) not only received widespread usage and citation upon publication but also maintained high levels of attention and application in subsequent years, indicating the sustained impact of their research findings. Scholars can explore such literature further to discover innovative insights.

## Distribution per journal

**Figure 3:**
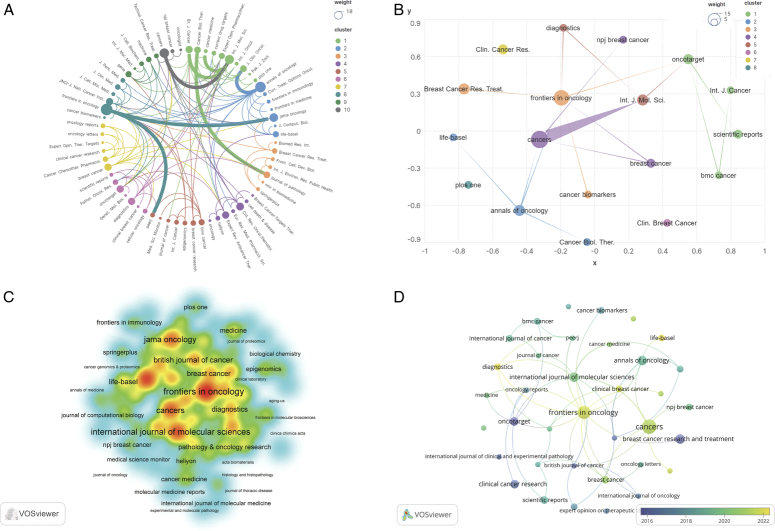
Network visualization of triple-negative breast cancer (TNBC) biomarker journals. (A) Collaborative network map of the largest journals. (B) Collaborative network map of journals with ≥4 publications. (C) Map of core journals in TNBC biomarkers. (D) Map of emerging journals in TNBC biomarkers.

We imported the Refworks data exported earlier into the VOSviewer software, selecting the analysis type and counting method (Citation-Sources), and found that a total of 127 journals contributed to TNBC biomarker research. However, the largest journal collaboration network included only 65 journals, indicating a lack of effective communication and interaction among journals (Fig. 3A). In the future, efforts should be made to enhance collaboration among journals, focusing on expanding the scale of journal collaboration networks, strengthening collaborative relationships to facilitate closer information sharing and academic exchange, thus advancing the in-depth development of TNBC biomarker research. To gain a clear understanding of the primary research directions and significant achievements in this field, attention was focused on journals with publication volumes of greater than or equal to 4 (Fig. 3B). The journal with the highest publication volume is *Cancers* (*N*=15), followed by *Frontiers in Oncology* (*N*=12), *Annals of Medicine* (*N*=6), *International Journal of Molecular Sciences* (*N*=6), and *Breast Cancer Research and Treatment* (*N*=6). *Annals of Oncology* has the highest total citation frequency (*N*=600). *Jama Oncology* has the highest average citation frequency per article (1 article, 453 citations). *Frontiers in Oncology*, *Annals of Oncology*, and *International Journal of Molecular Sciences* are core journals in TNBC biomarker research, playing leading and exemplary roles in this field (Fig. 3C). Currently, the research focus of core journals in the TNBC biomarker field is centered around exploring novel biomarkers^21,22^, investigating immune therapy-related markers^14,23^, assessing the potential value of microRNAs and long non-coding RNAs^24,25^, predicting genetic and epigenetic changes^26,27^, as well as developing therapeutic strategies targeting specific biomarkers^28,29^. Additionally, Frontiers in Oncology, Clinical Breast Cancer, Diagnostics, and Life-Basel are emerging journals with relatively high publication volumes in recent years (Fig. 3D). Through in-depth analysis of articles in these journals, we anticipate that future research focuses may revolve around circulating tumor DNA^30^, exploring biomarkers associated with immune therapy response^23,31^, investigating transcriptomics and gene fusion events^21^, understanding the mechanisms of miRNAs and lncRNAs^24^, as well as studying metabolic pathways and drug responsiveness^26,29^. These directions not only enhance understanding of the pathological mechanisms of TNBC but also facilitate the development of novel therapeutic approaches, ultimately achieving personalized treatment for TNBC patients.

**Figure 4:**
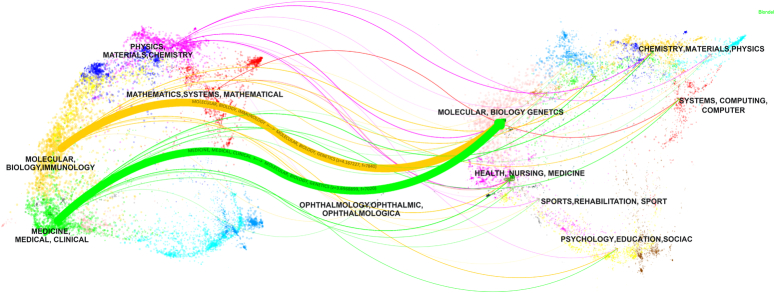
Double journal overlay map of triple-negative breast cancer biomarkers.

To further reveal the academic interaction and knowledge dissemination patterns among different journals, we utilized the Overlay Maps function in CiteSpace software to overlay dual maps of journals, thus exploring hidden interrelations of academic disciplines. Subsequently, we simplified the views based on the generated results (Z-score key), ultimately obtaining a simplified disciplinary coupling map (Fig. 4). It revealed two main citation pathways, namely MOLECULAR, BIOLOGY, IMMUNOLOGY → MOLECULAR, BIOLOGY, GENETICS (z=5.0958, f=3692); and MEDICINE, MEDICAL, CLINICAL → MOLECULAR, BIOLOGY, GENETICS (z=2.6693, f=2036). The graph indicates that key citations regarding TNBC biomarkers originate from MOLECULAR, BIOLOGY, GENETICS, implying significant potential and application significance of these disciplinary domains in TNBC biomarker research. Emerging researchers can focus on these domains and delve into their forefront research and methodologies to propel advancements in the TNBC biomarker field.

## Author and co-author analysis

**Figure 5:**
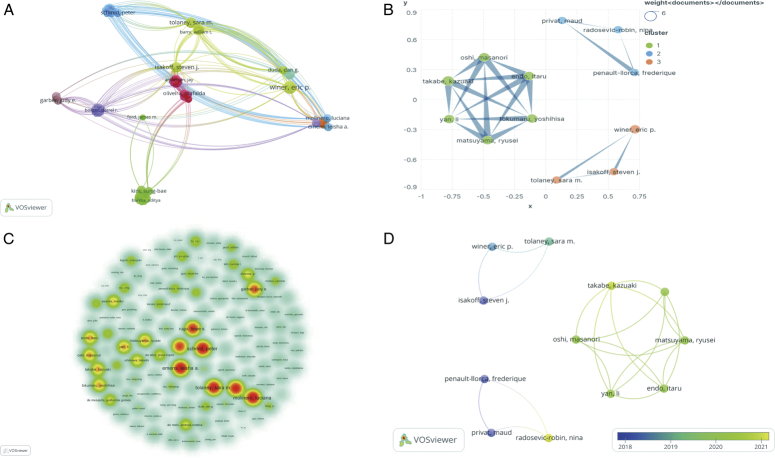
Visualization of the author network for triple-negative breast cancer (TNBC) biomarker papers. (A) Collaborative network map of the largest authors. (B) Collaborative network diagram of authors with ≥3 publications. (C) Core authors in TNBC biomarkers. (D) Emerging scholars in TNBC biomarkers.

To ensure that ambiguity in author abbreviations was avoided, we adjusted the abbreviated author information (e.g. modifying “Emens, LA” to “Emens, Leisha A.”). We found that a total of 1749 authors were involved in the TNBC biomarker study, which can be divided into eight author groups. The largest group contained 129 individuals, indicating that collaborative author research on TNBC biomarkers is still in its infancy (Fig. 5A). In Fig. 5A, the largest network was formed with Winer, Eric P. as the core, consisting of 48 individuals with a Total link strength of 52. Fig. 5B illustrates a graphical representation of author relationships for greater than or equal to 3 publications, which can be categorized into 3 main teams. the Privat, Maud team focuses on exploring the potential of microRNAs as biomarkers for triple-negative breast cancer^32–34^; the Oshi, Masanori team has made significant contributions to T cells and genes as biomarkers for triple-negative breast cancer^14,35–38^; and the Isakoff, Steven J.‘s team focuses on clinical research and hopes to explore relevant markers in clinical applications^8,39,40^. Takabe, Kazuaki has the most papers (*N*=5), followed by Winer, Eric P. (*N*=4), Endo, Itaru (*N*=4), Matsuyama, Ryusei (*N*=4), Oshi, Masanori Tokumaru (*N*=4), Yoshihisa (*N*=4), and Yan, Li (*N*=4). Authors with the highest total citations are Emens, Leisha A., Molinero, Luciana, Schmid, Peter (*N*=569), emphasizing the importance of using biomarkers in clinical practice^3,13^. Sarkar, Indrani averages the most citations per paper (*N*=453). Emens, Leisha A., Schmid, Peter, and Rugo, Hope S. are pivotal in this field (Fig. 5C), primarily validating the reliability of TNBC biomarkers through clinical trials. Takabe, Kazuaki and Radosevic-Robin, Nina are emerging scholars with significant recent publications (Fig. 5D). Takabe, Kazuaki’s team has proposed several promising biomarkers for predicting TNBC prognosis and treatment response. These markers include CD8 T Cell Score, Regulatory T Cell (Treg), ITPKC expression levels, Three-Gene Score, and Copy number alteration^14,35–38^. Radosevic-Robin, Nina’s team has explored microRNA, anti-EGFR antibodies, and Plasma Protein Profile as predictors of TNBC^34,41,42^. However, it should be noted that the application of biomarkers is still in the research stage and requires further validation and clinical application. Furthermore, integrating them with other clinical features and test results for comprehensive analysis can more accurately assess TNBC prognosis, treatment response, and individualized treatment strategies.

## Institutions

**Figure 6:**
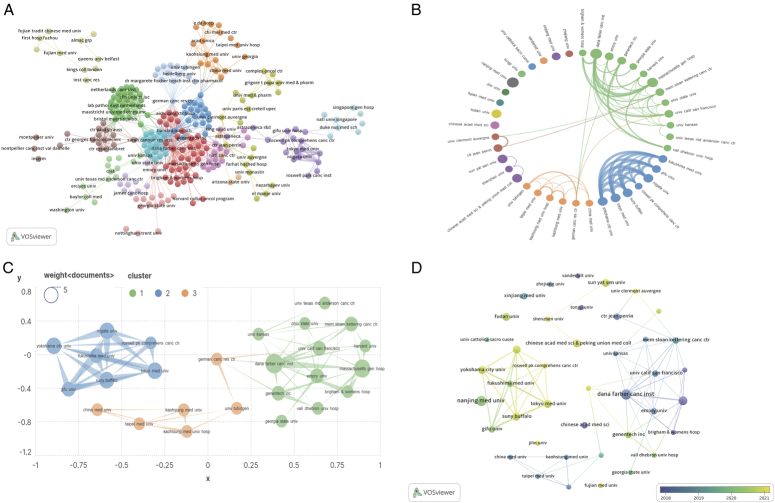
Visualization of Institutional networks in the triple-negative breast cancer (TNBC) biomarker field. (A) Institutional co-occurrence network map. (B) Collaborative network map of institutions with ≥3 publications. (C) Core institutions in TNBC biomarkers. (D) Emerging institutions in TNBC biomarkers.

To visually demonstrate the collaboration among institutions and research directions, we conducted an analysis of publishing institutions using VOSviewer in conjunction with Scimago Graphica software. The statistical results indicate that a total of 567 institutions participated in TNBC biomarker research, with the largest collaborative network consisting of 263 institutions organized into 16 main clusters (Fig. 6A). Dana Farber Cancer Institute constructed the largest collaborative network with a link count of 63 and a total link strength of 80, followed by Massachusetts General Hospital and Memorial Sloan Kettering Cancer Center. Dana Farber Cancer Institute and Nanjing Medical University had the highest number of publications (*N*=7), followed by Massachusetts General Hospital (*N*=6). The highest citation frequency was for Dana Farber Cancer Institute and Nanjing Medical University (*N*=1121), followed by Massachusetts General Hospital (*N*=929) and Johns Hopkins University (*N*=746). To utilize resources more efficiently and enhance the accuracy and practicality of the analysis results, we analyzed institutions with a publication count of greater than or equal to 3, discarding those with fewer publications (Fig. 6B). During the analysis of institutions with higher publication counts, we identified several prominent teams, centered around Fukushima Medical University, Dana Farber Cancer Institute, and Kaohsiung Medical University Hospital, conducting research in this field (Fig. 6C). Yokohama City University, SUNY Buffalo, and Tokyo Medical University have been prolific in recent years, with research focusing on exploring and validating immune cell infiltration, genomic variations, individual gene biomarkers, and multi-gene combination biomarkers^14,35–38^. These institutions have established collaborative relationships, engaging in research projects together, and providing mutual support and cooperation (Fig. 6D).Additionally, current research in this field is predominantly centered in universities. However, universities typically focus on basic research and academic exploration, which may pose challenges for the translation of TNBC biomarker research findings into practical applications.

## Countries/regions

**Figure 7:**
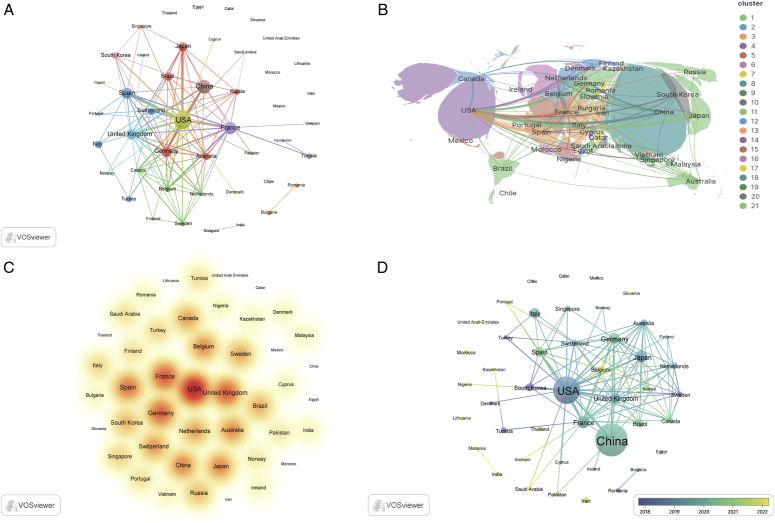
Triple-negative breast cancer (TNBC) collaborative network map of counties-regions in biomarkers. (A) Countries/regions cooperation network map. (B) Diagram of countries/regions map. (C) Core countries in TNBC biomarkers. (D) Emerging Countries in TNBC Biomarkers.

We analyzed the publication Countries/Regions using VOSviewer and Scimago Graphica software. To eliminate ambiguity in country names, we adjusted them according to the naming conventions of the Scimago Graphica software, for instance, changing “England”, “Scotland”, and “Northern Ireland” to “United Kingdom”. Researchers from 45 Countries/Regions participated in TNBC biomarker research, forming 21 major clusters (Fig. 7A, B). The USA has established the largest national collaboration network, followed by the United Kingdom, France, and Germany. China published the most articles (*N*=87), followed by the USA (*N*=65) and France (*N*=15). The USA has the highest citation count (*N*=2583), followed closely by China (*N*=1273) and France (*N*=921). The USA, France, and the United Kingdom are the core players in this field (Fig. 7C), holding prominent positions and notable achievements. Research in the USA focuses on novel predictive and prognostic biomarkers^26,28,43,44^, the application of biomarkers in treatment response assessment^20,39,40,45,46^, integrated studies of genetic and epigenetic biomarkers^19,47–51^, and immunological biomarkers^14,35,52,53^. Research in France on TNBC biomarkers encompasses immunotherapy^52,54^, drug treatments^26,55^, miRNA^32–34^, and other potential molecular markers^3,13,56,57^, aiming to elevate the diagnostic and therapeutic standards of TNBC, thus offering a plethora of choices and guidance for personalized patient care. Research in the United Kingdom on TNBC biomarkers primarily focuses on immune therapy-related biomarkers^3,13,52^, gene expression and molecular markers^51,58–61^, chemotherapy response-related biomarkers^62,63^, as well as biomarkers associated with treatment-related repair mechanisms^64^. The prominent contributions and characteristics of these four countries, along with their collaboration and communication, have collectively propelled the advancement of TNBC biomarker research and global cooperation. Through their efforts, we can better understand and address this complex disease. In recent years, countries such as Belgium, Iran, India, and Thailand have begun to pay attention to research in this field (Fig. 7D), primarily focusing on hot topics in TNBC biomarkers, including prognostic markers, gene expression profiling, immunohistochemical markers, demonstrating widespread interest and attention in TNBC biomarker research^10,26,52,54,65–68^.

## Research direction

Literature inclusion can be classified into 25 research themes according to WOS categories (Table 1). Among them, “ONCOLOGY” (*N*=123) stands as the most significant research direction. Following are “BIOCHEMISTRY & MOLECULAR BIOLOGY” (*N*=19) and “PATHOLOGY” (*N*=18). Theme analysis reveals the prominence of interdisciplinary research in the field of TNBC biomarkers. Involving multiple domains such as oncology, biochemistry, pathology, cell biology, genetics, etc. Biochemistry and molecular biology play pivotal roles in the identification and analysis of TNBC biomarkers. Pathology provides the foundation for the discovery of biomarkers. Genomic and genetic studies contribute to understanding the genetic characteristics of TNBC. Clinical translation and application prospects demonstrate the potential clinical value of this research field.

**Table 1:** WOS categories in papers on TNBC biomarkers.

WoS categories	Number
Oncology	123
Biochemistry & Molecular Biology	19
Pathology	18
Cell Biology	17
Medicine, Research & Experimental	13
Genetics & Heredity	12
Multidisciplinary Sciences	11
Medicine, General & Internal	10

TNBC, triple-negative breast cancer.

## Keywords co-occurrence, clusters and bursts

**Figure 8:**
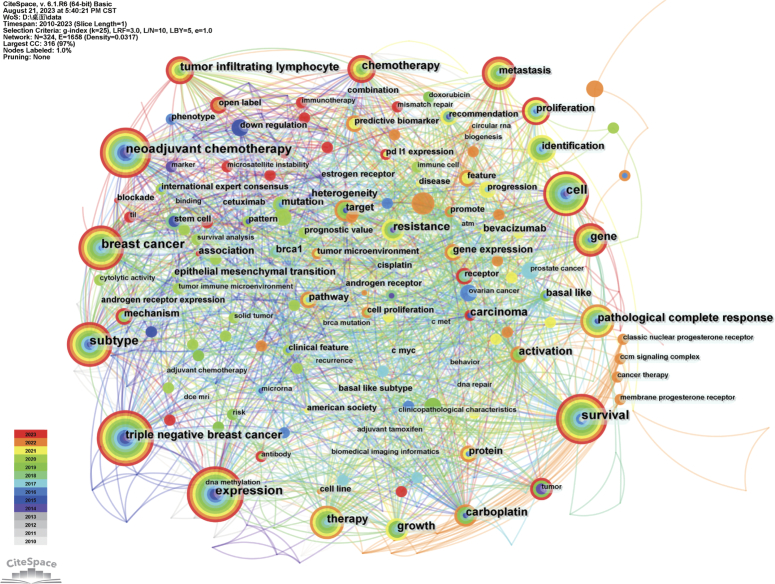
Network visualization of co-occurring keywords in triple-negative breast cancer biomarker domains.

Keyword analysis was conducted using CiteSpace software, with a total of 324 keywords included in the literature (Fig. 8). The most common terms include TNBC (*N*=122), expression (*N*=66), survival (*N*=45), breast cancer (*N*=30), neoadjuvant chemotherapy (*N*=30), and subtype (*N*=30). Keywords related to tumor characteristics and development include cell (*N*=27), tumor-infiltrating lymphocyte (*N*=21), metastasis (*N*=14), pathological complete response (*N*=14), proliferation (*N*=13), and carcinoma (*N*=12). The most common treatment methods and drugs include chemotherapy (*N*=22), therapy (*N*=16), carboplatin (*N*=13), resistance (*N*=9), and target (*N*=8). Keywords related to biomarker identification and expression include Identification (*N*=19), Gene (*N*=16), gene expression (*N*=8), and mutation (*N*=8). Common biomarkers include Epidermal growth factor receptor, c-KIT and cytokeratins, Androgen receptor, Tumor-infiltrating lymphocytes, *PD-L1/PD-1* expression, microRNAs, and long non-coding RNAs (Table 2).

**Table 2:** Biomarkers associated with TNBC.

Classification	Biomarkers	Function	References
Regulators of cell proliferation and migration and angiogenesis	Epidermal growth factor receptor (EGFR)	High EGFR expression is a poor prognostic factor for TNBC; a potential predictor of response to anti-EGFR therapy	^69–72^
	Fibroblast growth factor receptor (FGFR1̀ FGFR2)	Aberrant expression or mutation of FGFR may lead to proliferation, invasion and metastasis of TNBC.	^73–77^
	Angiogenic factor (VEGF\PDGF)	Angiogenic factors play an important role in angiogenesis and tumor development in TNBC.	^78–80^
	Ki-67	High Ki-67 expression in TNBC patients is usually associated with higher rates of tumor recurrence and malignancy. However, it has been shown that patients with high Ki-67 expression have a better pathological complete response after neoadjuvant chemotherapy.	^81–87^
	Androgen receptor (AR)	Patients with AR-positive TNBC usually have lower tumor recurrence rates and longer survival times. They are less sensitive to chemotherapy and more sensitive to AR inhibitors, PI3K inhibitors and their combinations.	^88–92^
	Human epidermal growth factor receptor 2(HER2)	In early-stage triple-negative breast cancer, low HER2 status was positively associated with DFS and OS, but not significantly different from pCR, and may serve as a potential prognostic marker for individualized treatment decisions.	^93–97^
RNA biomarkers associated with TNBC	MicroRNAs	MiRNAs play a broad role in the early diagnosis of TNBC, prognostic monitoring and breast cancer including chemotherapy, radiotherapy and immunotherapy	^98–102^
	Long non-coding RNAs (lncRNA)	lncRNAs play an important role in the characteristics of TNBC genesis, proliferation, migration, relapse, and chemoresistance.	^15,103–106^
DNA biomarkers associated with TNBC	TP53 mutation	TP53 gene mutation is associated with tumor invasiveness, prognosis and chemotherapy resistance	^107–111^
	BRCA1/2 germline mutation	BRCA mutations play an important role in the pathogenesis of TNBC. The role of BRCA status as an independent predictive biomarker in neoadjuvant therapy in the TNBC population is unclear, and studies have shown conflicting results.	^112–116^
	PIK3CA	PIK3CA mutations may be associated with poor prognosis and tumor recurrence in TNBC.	^117,118^
	PTEN loss	Loss of PTEN has been shown to be associated with aggressiveness, risk of recurrence and poor prognosis in TNBC. Greater sensitivity to combination therapy with PI3K and androgen receptor inhibitors	^119–123^
	Circulating tumor DNA (ctDNA)	Efficient and non-invasive detection of genetic mutations in TNBC patients, including variations in targets of drug action, contributes to the development of personalized treatment regimens and improves clinical benefit.	^124–126^
Immunobiomarkers associated with TNBC	Tumor-infiltrating lymphocytes (TILs)	Higher levels of TILs infiltration in TNBC patients are usually associated with a better prognosis, including a better response to immunotherapy and predictive significance in predicting relapse and overall survival.	^127–130^
	Programmed death-ligand 1 (PD-L1)	The expression of PD-L1 in TNBC is closely related to tumor immune escape, malignant degree and treatment response.	^131–134^
	CTLA-4	Upregulation of CTLA-4 in TNBC is a key mechanism of immune escape and may affect the efficacy of immunotherapy. Blocking CTLA-4 with inhibitors such as Ipilimumab may activate the immune response against tumor cells, offering hope for enhanced therapeutic efficacy.	^135–139^
	Adoptive cellular immunotherapy (CD8 T Cells)+	CD8 T cells and their activity and concentration can influence tumor development, prognosis and immunotherapeutic efficacy.	^140–142^

TNBC, triple-negative breast cancer.

**Figure 9:**
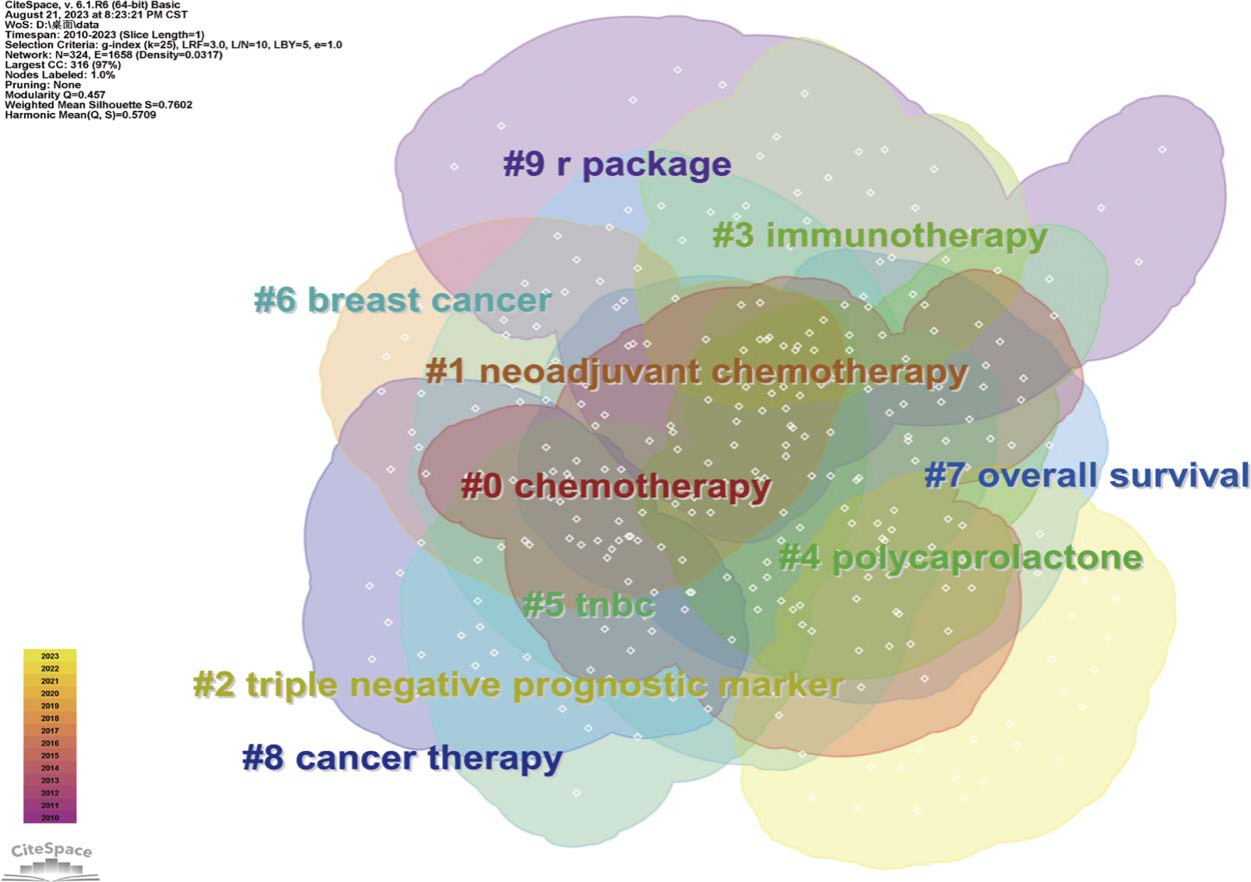
Network visualization of triple-negative breast cancer biomarker keyword clustering.

**Figure 10:**
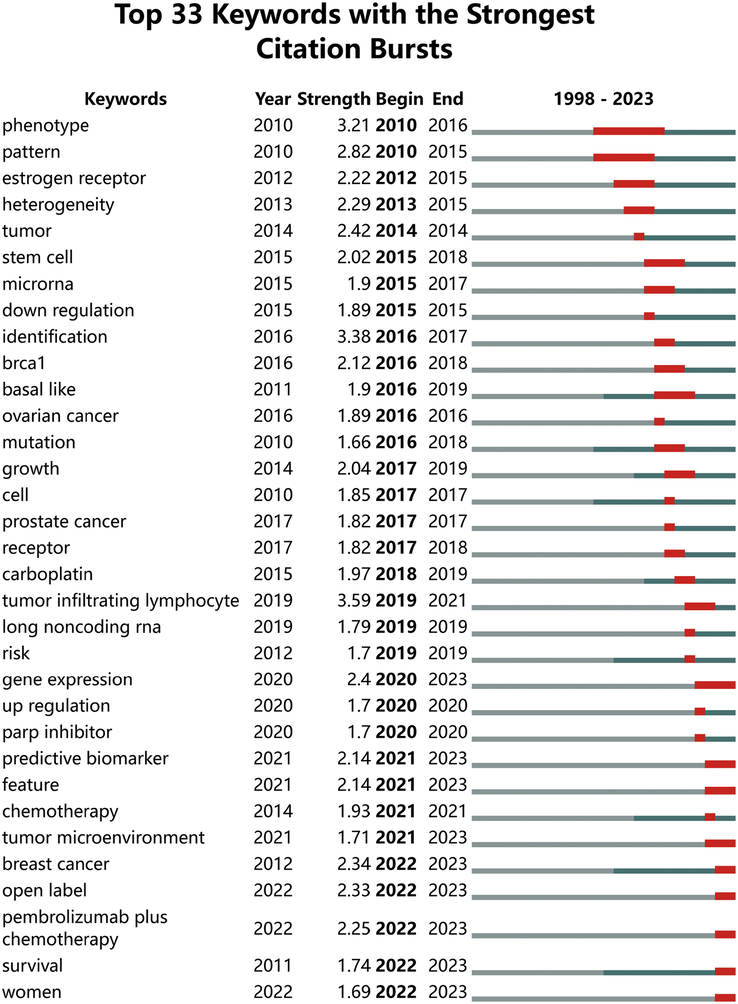
Most explosive keywords for triple-negative breast cancer biomarker citations.

**Figure 11:**
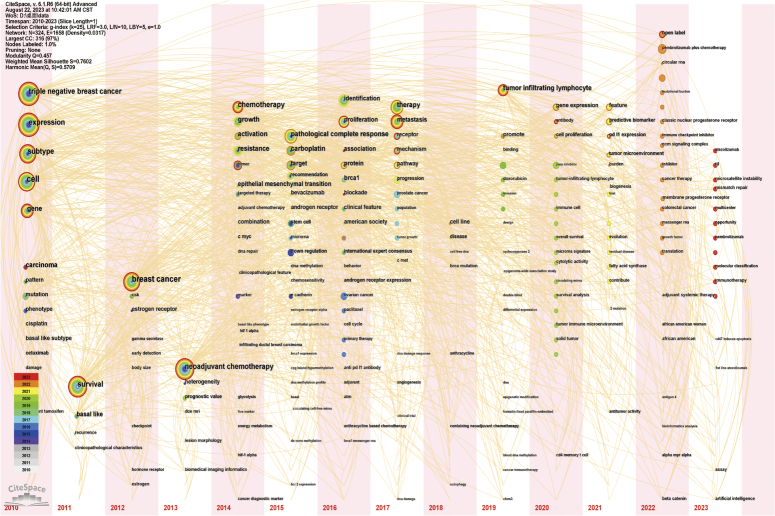
Timeline mapping of triple-negative breast cancer biomarker domain keywords.

**Figure 12:**
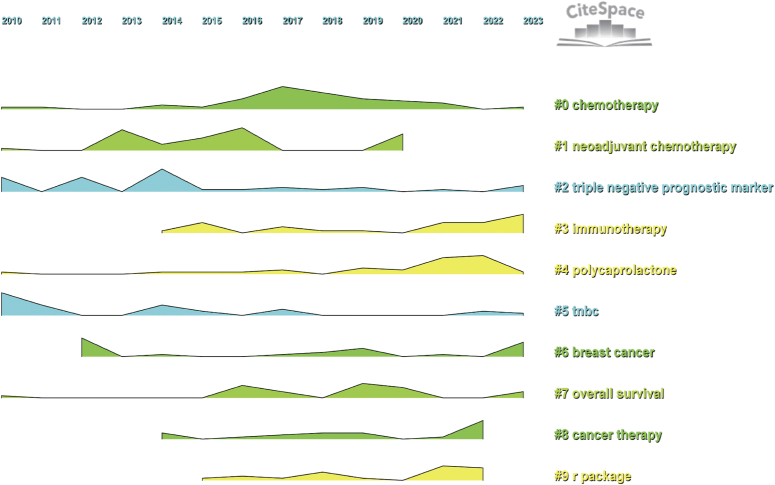
Timeline mapping for triple-negative breast cancer biomarker keyword clustering.

To better illustrate the research hotspots in this field, clustering analysis of TNBC biomarker keywords was conducted based on keyword co-occurrence using the Log-Likelihood Ratio method (Fig. 9, Table 3). Red and orange clusters primarily involve biomarkers associated with TNBC chemotherapy patients. Yellow cluster mainly relates to prognostic biomarkers of TNBC. The green cluster focuses on biomarkers in the immunotherapy of TNBC. Light blue cluster primarily focuses on the mechanisms of development, treatment strategies, and prognostic prediction of breast cancer and TNBC. Blue cluster mainly focuses on evaluating epidemiological surveys and risk biomarkers in TNBC patients. Purple cluster primarily focuses on data analysis and modeling using R packages, analyzing copy number variations in the genome, identifying differentially expressed genes, and exploring potential therapeutic drugs and methods. Additionally, we conducted keyword burst analysis (Fig. 10), timeline analysis (Fig. 11), and clustered timeline analysis (Fig. 12). From the figures, we observe that keywords such as phenotype, pattern, gene, and cell have consistently garnered scholars’ attention since 2010. Over the past three years, researchers in the TNBC biomarker field have primarily focused on gene expression, open-label studies, tumor microenvironment, and proteins. Additionally, scholars are increasingly interested in the discovery and application of predictive biomarkers, which can enhance patient treatment outcomes, reduce wastage of ineffective treatments, minimize unnecessary side effects and costs. These areas also represent key directions for future research.

**Table 3:** Keyword cluster analysis of TNBC biomarker papers.

Cluster	Size	Silho-uette	Mean year	Label (LLR)
0	42	0.634	2017	Chemotherapy (9.31, 0.005); cyclophosphamide (4.09, 0.05); ovarian cancer (4.09, 0.05); paclitaxel (4.09, 0.05); serum her2 (4.09, 0.05)
1	39	0.738	2015	Neoadjuvant chemotherapy (14.13, 0.001); treatments (5.98, 0.05); breast cancer (3.82, 0.1); survival (3.53, 0.1); immunotherapy (3.53, 0.1)
2	36	0.84	2014	Triple-negative prognostic marker (5.6, 0.05); lncrnas (5.6, 0.05); fasn (5.6, 0.05); parp1 (5.6, 0.05); oncogenes (5.6, 0.05)
3	30	0.843	2019	Immunotherapy (10.78, 0.005); molecular classification (9.86, 0.005); immune checkpoint inhibitors (6.22, 0.05); artificial intelligence (4.92, 0.05); DNA damage repair (4.92, 0.05)
4	29	0.771	2019	Polycaprolactone (4.92, 0.05); selenoproteins (4.92, 0.05); pim-1 (4.92, 0.05); pi3k inhibitor (4.92, 0.05); therapeutics (4.92, 0.05)
5	29	0.766	2013	TNBC (5.63, 0.05); expression (4.75, 0.05); egfr (4.26, 0.05); tumor suppressor (3.88, 0.05); gene expression signature (3.88, 0.05)
6	25	0.717	2017	Breast cancer (13.86, 0.001); doxorubicin (5.85, 0.05); macrophage (5.85, 0.05); immune-related toxicity (5.85, 0.05); long-term survival (5.85, 0.05)
7	25	0.749	2018	Overall survival (5.91, 0.05); biomarker discovery (4.76, 0.05); risk biomarker (4.76, 0.05); clinical oncology/college (4.76, 0.05); chk1 (4.76, 0.05)
8	22	0.801	2019	Cancer therapy (12.89, 0.001); mifepristone (12.89, 0.001); ccm signaling complex (12.89, 0.001); tumorigenesis (12.89, 0.001); progesterone (12.89, 0.001)
9	22	0.745	2019	R package (5.74, 0.05); copy number alteration (5.74, 0.05); differentially expressed genes (5.74, 0.05); metergoline (5.74, 0.05); neoadjuvant chemotherapy therapy (5.74, 0.05)

TNBC, triple-negative breast cancer.

### Co-cited articles and co-cited reference cluster analysis

**Figure 13:**
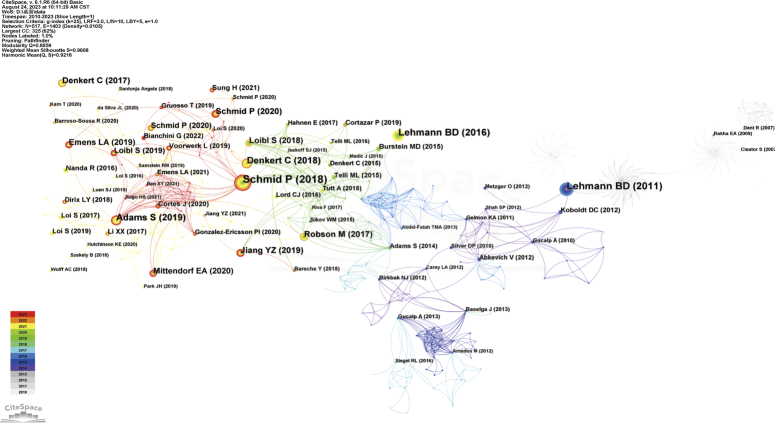
Co-citation visualization analysis of triple-negative breast cancer biomarker literature.

Co-citation analysis of literature is a method used to reveal interconnections among documents by detecting other documents that cite the same article, thereby determining their relationships^143^. Conducting Co-citation analysis on literature in the TNBC biomarker field can help us understand the forefront advancements, primary research directions, and highly impactful documents within the field. We utilized CiteSpace software for co-citation analysis on the included literature (Fig. 13). Through literature co-citation analysis, we can observe that research on TNBC biomarkers can be roughly divided into three stages: before 2011, research on TNBC biomarkers primarily focused on identifying different subtypes of TNBC and establishing preclinical models to select targeted treatment approaches. Researchers successfully categorized TNBC into different subtypes by analyzing the gene expression profiles and biological characteristics of TNBC samples, and explored the associated molecular mechanisms^144^. From 2012 to 2017, following Brian D Lehmann’s significant discoveries, researchers began studying biomarkers for different TNBC subtypes and started focusing on treatment strategies and prognosis assessment^144^. Clinical trial results indicate that neoadjuvant chemotherapy can improve treatment outcomes for TNBC patients. Consequently, researchers began exploring TNBC biomarkers in the context of neoadjuvant chemotherapy^127,131,145^. From 2018 to present, research focus on TNBC biomarkers has further expanded, emphasizing precision medicine and personalized treatment strategies. Researchers have shifted their focus towards new biomarkers and technologies, such as circulating tumor DNA, bioinformatics, and transcriptome analysis, to identify potential therapeutic targets and strategies.

**Figure 14:**
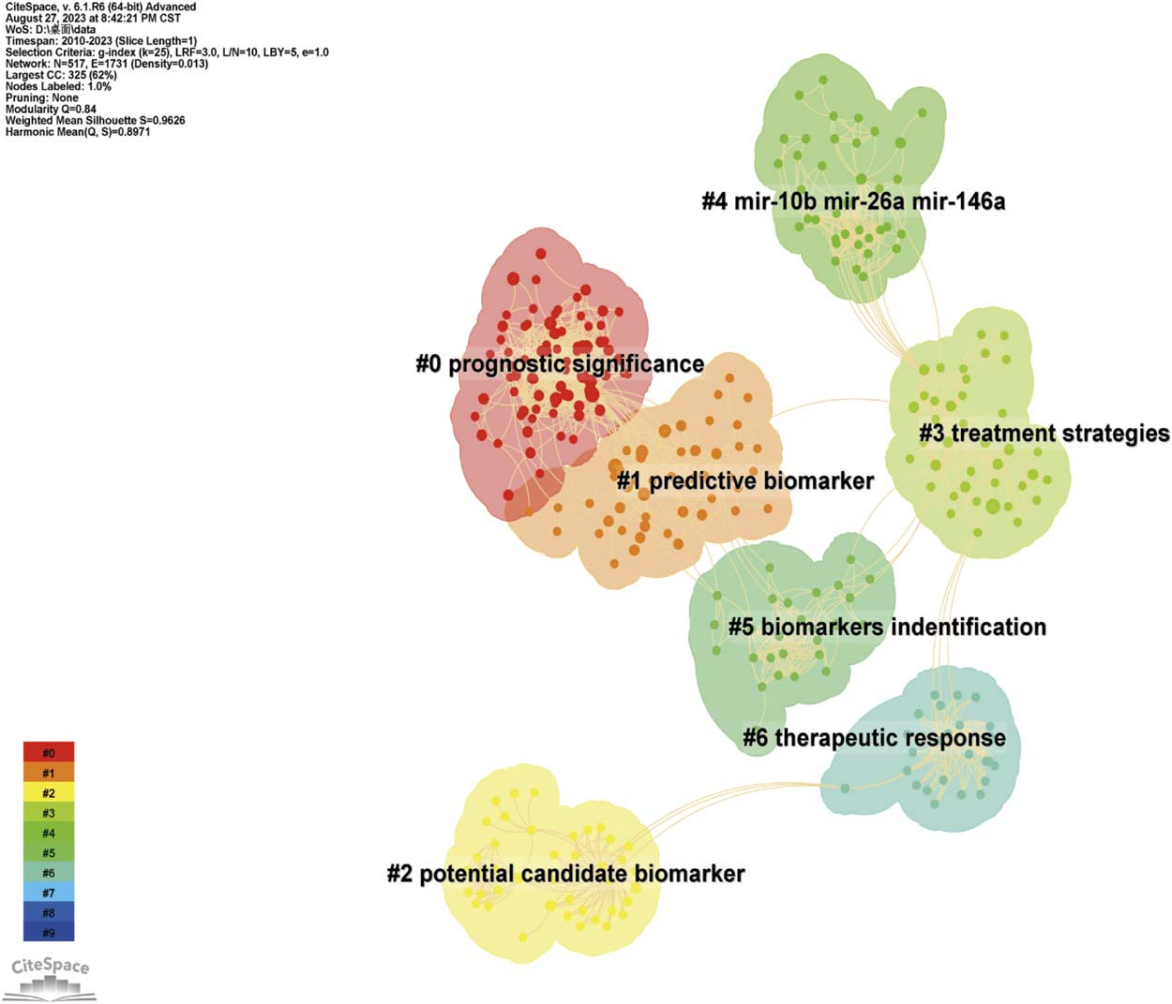
Visualization of triple-negative breast cancer biomarker co-citation literature clustering.

We performed clustering of references (Fig. 14, Table 4) and identified 7 major research domains in TNBC biomarkers: Cluster 0 focuses on the prognostic significance of TNBC biomarkers and current testing methods, including prognostic assessment, biomarker research, phase II clinical trials, and single-cell multi-omics, aiming to enhance prognostic evaluation and personalized treatment for TNBC patients. Cluster 1 focuses on predictive biomarkers and patient stratification research, encompassing predictive biomarker evaluation, patient stratification, mutation status, etc., aiming to discover biomarkers predicting treatment response and prognosis in TNBC patients for personalized treatment strategies. Cluster 2 focuses on potential candidate biomarkers and the heterogeneity of TNBC, with research concentrating on the evaluation of candidate biomarkers, disease heterogeneity, Notch-4, etc., which contributes to understanding the molecular characteristics and subtypes of TNBC. Cluster 3 primarily focuses on treatment strategies and TNBC subtypes research, including treatment strategy evaluation, TNBC subtype analysis, genomics, etc., aiming to explore treatment strategies for TNBC, improve clinical decisions, and predict prognosis for TNBC patients. Cluster 4 focuses on research about miRNA and other epigenetic changes, including miRNA expression, other epigenetic changes, potential biomarkers, etc., aiming to reveal the roles of miRNA and other epigenetic changes in TNBC development and find new biomarkers. Cluster 5 focuses on research into biomarker identification and the sensitivity of TNBC to chemotherapy, covering aspects such as biomarker identification, assessment of chemotherapy sensitivity, and drug response in TNBC patients, contributing to the discovery of new biomarkers and predicting the sensitivity of TNBC patients to chemotherapy. Cluster 6 focuses on the research of treatment response and imaging-based detection methods, including aspects such as treatment response assessment, imaging-based biomarkers, which can aid in evaluating the treatment response of TNBC patients and exploring imaging-based biomarkers.

**Table 4:** Reference clustering of TNBC biomarker papers.

Cluster	Size	Silho-uette	Mean year	Label (LLR)
0	76	0.956	2019	Prognostic significance (26.87, 1.0E-4); current testing method (20.09, 1.0E-4); biomarker study (17.84, 1.0E-4); phase ii (17.84, 1.0E-4); single-cell multi-omics (15.59, 1.0E-4)
1	49	0.919	2016	Predictive biomarker (30.98, 1.0E-4); prognostic significance (15.3, 1.0E-4); mutational status (13.08, 0.001); promising predictive biomarker (13.08, 0.001); patient stratification (11.79, 0.001)
2	42	1	2008	Potential candidate biomarker (19.54, 1.0E-4); heterogeneity (19.54, 1.0E-4); notch-4 biomarker expression (9.63, 0.005); triple-negative breast cancer (1.86, 0.5); triple-negative breast cancer (1.24, 0.5)
3	39	0.927	2011	Treatment strategies (36.69, 1.0E-4); triple-negative breast cancer subtype (36.69, 1.0E-4); cancer genomics (32.03, 1.0E-4); predicting prognosis (27.4, 1.0E-4); anthracycline-based adjuvant chemotherapy (27.4, 1.0E-4)
4	37	0.985	2012	mir-10b mir-26a mir-146a (34.78, 1.0E-4); other epigenetic change (27.69, 1.0E-4); triple-negative v (20.66, 1.0E-4); potential biomarker (20.66, 1.0E-4); mir-153 expression (20.66, 1.0E-4)
5	27	0.975	2013	Biomarkers indentification (25.36, 1.0E-4); triple-negative breast cancer chemosensitivity (25.36, 1.0E-4); current advance (25.36, 1.0E-4); triple-negative breast cancer patient (11.27, 0.001); protein expression analysis (8.31, 0.005)
6	25	0.976	2010	Therapeutic response (11.84, 0.001); dynamic contrast-enhanced MRI-based biomarker (11.84, 0.001); triple-negative breast cancer (0.47, 0.5); triple-negative breast cancer (0.45, 1.0); metastatic triple-negative breast cancer (0.17, 1.0)

TNBC, triple-negative breast cancer.

**Figure 15:**
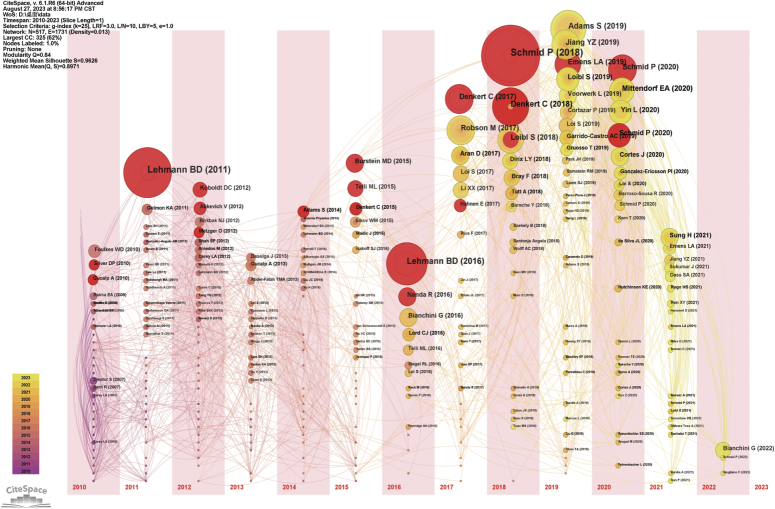
Evolution and maximum citation intensity analysis of co-cited literature for triple-negative breast cancer biomarkers.

We employed Citespace software to identify the evolution process and burst intensity of co-cited papers included in the literature over the past decade (Fig. 15, Table 5). Through burst analysis of references in articles on TNBC biomarkers, we found that the early focus was on exploring factors related to race, subtypes, and survival rates^146^. Over time, researchers began to seek treatment selection methods and analyze the prognostic value of different biomarkers in treating this subtype of breast cancer^147,148^. Studies on treatment response, especially in neoadjuvant therapy, indicate their significant impact on the long-term survival of TNBC patients^149,150^. Furthermore, studies on molecular characteristics have revealed specific biomarkers of basal-like TNBC, further advancing the understanding of molecular characterization of different subtypes of triple-negative breast cancer^151^. From 2015 to 2023, the research focus shifted to the evaluation of tumor-infiltrating lymphocytes (TILs), with related guidelines and reviews widely cited^127^. This indicates the gradually recognized prognostic role of TILs in TNBC, becoming a current research focus. On the other hand, research on immune checkpoint inhibitors (ICIs), especially atezolizumab and pembrolizumab, in TNBC treatment has rapidly increased, demonstrating their importance as potential therapies^3,13,128,131,152,153^. Research on PARP inhibitors has also shown significant growth, especially for TNBC patients with BRCA mutations or other homologous recombination repair defects^145,154,155^. Considering the recent high burst citation intensity of literature related to TILs and ICIs, it is foreseeable that immunorelated biomarkers and immunotherapy literature may have higher citation frequencies in the future. Similarly, research related to PARP inhibitors, especially studies targeting *BRCA* mutation carriers, also suggests that papers in this field may continue to receive high attention.

**Table 5:**
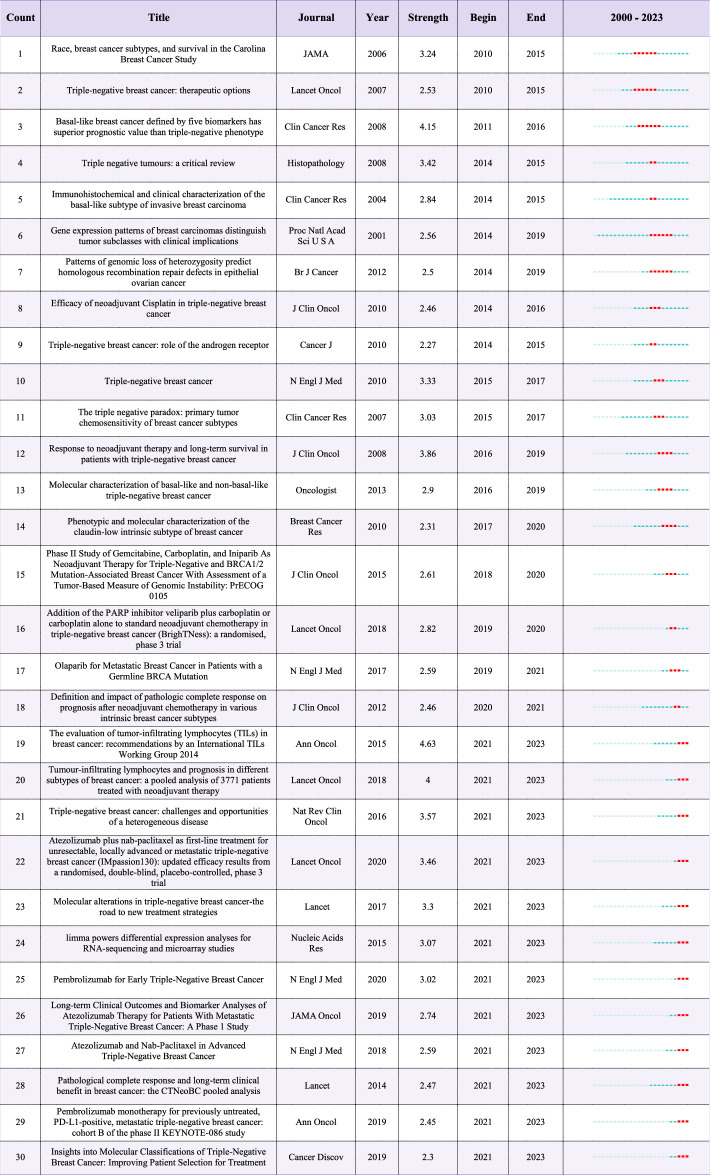
Top 30 references with the strongest citation bursts.

## The top 10 co cited articles related to TNBC biomarkers

Highly cited literature serves as influential and significant works that can be cited as classical references in the field^143^. We conducted co-citation analysis on 217 TNBC biomarker literature and simultaneously selected 10 highly co-cited literature relevant to TNBC biomarkers (Table 6). These articles highlight the complexity of TNBC, focusing on identifying prognostic and predictive biomarkers that can guide personalized therapy for TNBC patients. A recurring theme in these key studies is TILs as biomarkers for predicting treatment response and prognosis^127,129,156,157^. For instance, articles authored by Adams and colleagues and Loi and colleagues emphasize the role of TILs in enhancing neoadjuvant chemotherapy efficacy and serving as reliable independent prognostic markers. The emphasis on TILs highlights a shift in focus of TNBC research towards immunotherapy and the tumor microenvironment. Another core focus is on identifying and characterizing TNBC subtypes through gene expression profiling, as demonstrated by Lehmann *et al.* ^144^. This groundbreaking study not only categorizes TNBC into six distinct subtypes but also paves the way for targeted therapies by identifying subtype-specific vulnerabilities. Similarly, the identification of predictive biomarkers based on chemotherapy and novel agents, as emphasized in the study by Emens *et al.* ^13^, highlights the significance of immune biomarkers such as *PD-L1* expression in determining the efficacy of immune checkpoint inhibitors in metastatic TNBC treatment, revealing the crucial importance of considering immune biomarkers (such as *PD-L1* expression) in personalized approaches. Furthermore, studies on the prognostic value of basal-like markers over triple-negative phenotype, such as that by Cheang *et al.* ^148^, demonstrate that basal-like breast cancer defined by five biomarkers has superior prognostic value compared to the triple-negative phenotype, and expanded immunohistochemical marker panels can more accurately predict survival outcomes in breast cancer patients. This further supports the notion that TNBC is a collection of several diseases rather than a single entity, necessitating targeted treatment approaches based on specific biomarker profiles. The integration of laboratory-based biomarkers into clinical practice underscores the importance of interdisciplinary approaches. As it requires not only identifying biomarkers but also effectively interpreting these markers in a clinical context, enabling oncologists to provide the most appropriate targeted therapy for patients. This approach holds promise in improving outcomes for TNBC patients traditionally limited in treatment options due to the lack of hormone receptors and *HER2* expression.

**Table 6:** The top 10 co cited articles related to TNBC biomarkers.

Year	Citation	First author	Journal	Title
2011	^144^	Lehmann *et al*.	J Clin Invest	Identification of human triple-negative breast cancer subtypes and preclinical models for selection of targeted therapies
2014	^129^	Adams *et al*.	J Clin Oncol	Prognostic value of tumor-infiltrating lymphocytes in triple-negative breast cancers from two phase III randomized adjuvant breast cancer trials: ECOG 2197 and ECOG 1199
2018	^127^	Denkert *et al*.	Lancet Oncol	Tumour-infiltrating lymphocytes and prognosis in different subtypes of breast cancer: a pooled analysis of 3771 patients treated with neoadjuvant therapy
2015	^156^	Salgado *et al*.	Ann Oncol	The evaluation of tumor-infiltrating lymphocytes (TILs) in breast cancer: recommendations by an International TILs Working Group 2014
2019	^157^	Emens LA	JAMA Oncol	Long-term Clinical Outcomes and Biomarker Analyses of Atezolizumab Therapy for Patients With Metastatic Triple-Negative Breast Cancer
2010		Silver DP	J Clin Oncol	Efficacy of Neoadjuvant Cisplatin in Triple-Negative Breast Cancer
2008	^148^	Cheang *et al*.	Clin Cancer Res	Basal-like breast cancer defined by five biomarkers has superior prognostic value than triple-negative phenotype
2019	^7^	Loibl *et al*.	Ann Oncol	A randomised phase II study investigating durvalumab in addition to an anthracycline taxane-based neoadjuvant therapy in early triple-negative breast cancer: clinical results and biomarker analysis of GeparNuevo study
2019	^158^	Loi *et al*.	J Clin Oncol	Tumor-Infiltrating Lymphocytes and Prognosis: A Pooled Individual Patient Analysis of Early-Stage Triple-Negative Breast Cancers
2021	^13^	Emens *et al*.	J Natl Cancer Inst	Atezolizumab and nab-Paclitaxel in Advanced Triple-Negative Breast Cancer: Biomarker Evaluation of the IMpassion130 Study

TNBC, triple-negative breast cancer.

In summary, these articles collectively delineate key trends in TNBC research, including a nuanced understanding of TNBC subtypes, the prognostic and therapeutic relevance of TILs, the emerging role of immunotherapy, and the importance of biomarker-driven treatment strategies. With advancements in genomic and immunologic analytical techniques, achieving personalized therapeutic goals for TNBC becomes increasingly feasible. However, challenges persist in translating these promising biomarkers into routine clinical practice, necessitating ongoing collaboration among researchers, clinicians, and patients.

## Discussion

In this study, we utilized bibliometric tools such as CiteSpace, VOSviewer, and Scimago Graphica to analyze the literature on TNBC biomarkers, aiming to investigate global research trends and key findings. Through retrieving and analyzing a large number of relevant literature, we have drawn several key conclusions as follows: Firstly, we observed a significant growth trend in TNBC biomarker research in recent years. With the increasing demand for personalized treatment of TNBC, academic attention in this field is also rising. This indicates the gradually recognized importance of biomarkers in TNBC research, and the related studies are widely conducted globally. Data analysis revealed a total of 1749 authors contributing to TNBC biomarker research. Emens, Leisha A., Schmid, Peter, and Rugo, Hope S. stand as prominent representatives in this field, while Takabe, Kazuaki, and Radosevic-Robin, Nina emerge as notable emerging scholars with significant publications in recent years. A total of 127 journals published research on TNBC biomarkers. *Frontiers in Oncology, Annals of Oncology, and International Journal of Molecular Sciences* are core journals in TNBC biomarkers, while Clinical Breast Cancer, Diagnostics, and Life-Basel are emerging journals with relatively high publication volumes in recent years. Productive research institutions have established relatively stable collaborative groups, with Fukushima Med Univ, Dana Farber Canc Inst, and Kaohsiung Med Univ Hosp as the core contributors in this field. Yokohama City Univ, Suny Buffalo, and Tokyo Med Univ are institutions with higher publication rates in recent years. National analysis reveals that currently developed countries (USA, France, United Kingdom, etc.) hold a dominant position in this field, which is closely related to their advantages in research funding, research institutions, technological level, clinical resources, and international collaboration. In recent years, with the deepening of international scientific cooperation and the lowering of research barriers due to technological advancements, developing countries (Iran, India, Thailand, etc.) are expected to play a more significant role in this field in the future. Analysis of keywords and cited references reveals researchers’ interests and research directions in finding new biomarkers, exploring TNBC subtypes, evaluating treatment strategies, and predicting patient prognosis. Additionally, through the analysis of keyword bursts and keyword temporal patterns, we observe that some keywords such as phenotype, pattern, gene, and cell have been consistently focused on by scholars since 2010. In recent years, researchers have shown increased interest in keywords such as gene expression, open-label, tumor microenvironment, and protein, and have shown significant interest in the discovery and application of predictive biomarkers. Finally, through clustering analysis of references, we found that research on TNBC biomarkers primarily focuses on TNBC subtypes, prognosis, treatment strategies, and neoadjuvant chemotherapy. The aim of these studies is to improve treatment outcomes for TNBC patients, personalize treatments, and provide better prognostic evaluations. In conclusion, our research findings reveal the global trends and key directions in TNBC biomarker research. These results are of significant importance in advancing the field, fostering collaboration and knowledge sharing, and providing more accurate and personalized approaches for the diagnosis, treatment, and prognosis of TNBC patients.

In this study, we analyzed core authors, institutions, countries, keywords, and co-cited literature in the field of TNBC biomarkers. Our findings indicate that current research primarily focuses on immune checkpoint markers (such as *PD-L1* expression and levels of immune cell infiltration), microenvironment-associated markers (like tumor-associated lymphocytes and tumor-associated macrophages), circulating tumor DNA (*ctDNA*), metabolic markers (including metabolites and metabolic enzymes), and genomic markers (mutations, gene expression, and genomic structural variants). These research focal points contribute to a deeper understanding of TNBC’s molecular characteristics and biological processes, thereby providing more precise biomarkers for its diagnosis, treatment, and prognosis.

By analyzing emerging authors, institutions, countries, highly cited, and recently highly used literature in this field, we anticipate future TNBC biomarker research to pivot towards multi-omics integration (incorporating genomics, transcriptomics, proteomics, and metabolomics), detailed exploration of the microenvironment (including different cell types, cytokines, and cell-cell interactions), targeted therapy biomarkers (associated with TNBC drug sensitivity and resistance), liquid biopsy applications (such as *ctDNA*), utilization of machine learning and artificial intelligence (including data mining and predictive modeling), and personalized treatment strategies. We encourage young researchers to engage in interdisciplinary learning and collaboration, stay abreast of new technologies and methodologies, enhance their data analysis skills, and remain informed about the latest research trends to effectively address the challenges and opportunities in the field of TNBC biomarkers.

## Limitations

### Data sources

Our study is mainly based on published papers in the Web of Science (WOS) Core Collection database. This may result in some unpublished studies or grey literature not being included, which may affect the comprehensiveness and accuracy of the results.

### Data analysis

Although we used a bibliometric approach for our analysis, this method of analysis has its own limitations. We did not have access to raw data from the literature and had to rely on the information provided in the papers. In addition, our analyses were limited by the keywords chosen and the time frame.

Interpretation of data: our interpretation of the number of publications, citations and keywords may be somewhat subjective. Therefore, different researchers may reach different conclusions.

## A diagnostic algorithm for TNBC

TNBC is a special type of breast cancer in which the tumor cells do not contain estrogen receptor (ER), progesterone receptor (PR) and HER2. This makes conventional hormone therapy and HER2 targeting ineffective for TNBC, making diagnostic accuracy especially important. Below is an overview of a diagnostic algorithm for TNBC based on current medical knowledge and practice:

### Initial assessment and screening

1. Initial telemedicine assessment: initial history collection and risk assessment via telemedicine platform.
2. Self-detection instruction: educating patients on breast self-examination through online platforms and mobile applications.
3. High-risk screening procedures: Early and frequent specialised screening is recommended for individuals with a family history or other high-risk factors.

This stage focuses primarily on risk assessment and early detection, with relatively limited use of biomarkers. However, for high-risk groups, testing for genetic markers associated with breast cancer risk may be considered. For example genetic mutation testing (*BRCA1* and *BRCA2*) is used to assess the risk of familial breast cancer. In addition, other genetic mutations, such as *PALB2* and *TP53*, have been associated with increased breast cancer risk.

### Accurate diagnosis

1. AI-assisted imaging: AI technology is applied to analyze mammograms, ultrasound and MRI images to improve the accuracy of detecting early tumors.
2. Rapid histological assessment: accelerated histological diagnosis using rapid biopsy and immunohistochemical testing to ensure rapid determination of TNBC status.
3. Genetic and biomarker analysis: Comprehensive genetic and biomarker testing is performed on all patients with confirmed TNBC to guide subsequent treatment selection.

At this stage, biomarker testing is critical to confirm TNBC status and the biology of the disease. For example, immunohistochemistry (IHC) was used to detect ER(−), PR(−) and HER2(−) protein expression in patient tumor samples; tumor proliferation indices were assessed by IHC methods, and high expression of *Ki-67* was associated with more aggressive tumor growth rates.

### Staging and personalized treatment planning

1. Molecular typing and staging: precise staging and molecular typing based on the results of genetic and biomarker analysis.
2. Multidisciplinary team (MDT) discussion: medical experts from different specialties are assembled through virtual meetings to develop and adapt a personalized treatment plan.

After a TNBC diagnosis has been established, biomarkers play an important role in disease staging, prognostic assessment and treatment planning. For example, the expression of *PDL1* protein is detected by IHC as a potential predictive marker of immunotherapy response; the level of TILs can be used as a predictor of immunotherapy response and correlates with prognosis.

### Treatment monitoring and adjustment

1. Treatment response monitoring: use advanced imaging and molecular marker techniques to monitor treatment response.
2. Dynamic treatment adjustment: Timely adjustment of the treatment plan according to the treatment effect and disease changes.

In the course of treatment, biomarker monitoring is crucial for assessing treatment response, adjusting the treatment plan and predicting disease progression. For example detection of ctDNA through blood samples can be used to monitor treatment response and early detection of recurrence or metastasis; the number and characteristics of CTCs can be used as an indicator of treatment efficacy and help to implement a more personalized treatment plan.

### Ongoing updates and patient education

During the course of treatment, clinicians should conduct regular mental health assessments of patients to ensure that necessary psychological support and interventions can be provided in a timely manner. In addition, doctors should keep abreast of the latest scientific research and technological advances, regularly update the diagnosis and treatment process of TNBC, and help patients and their families to gain a deeper understanding of disease-related diagnostic and therapeutic information as well as daily life management strategies through the provision of personalized educational materials and the organization of workshops.

## Ethical approval

Not applicable. Our article does not deal with humans and is an econometric analysis of the literature within the original database.

## Consent

Not applicable. Our article does not deal with humans and is an econometric analysis of the literature within the original database.

## Source of funding

Qilu Medical School Traditional Chinese Medicine Academic Inheritance Project (2020]132). The National Natural Science Fund of China provided funding for this study (No. 82174491).

## Author contribution

W.X.X. conducted the study, produced the first draft, and updated it for this research on medical insurance. L.X.H., Y.W.Y., and D.T.T. conducted the literature search, retrieval, and data collection. L.X.H. and Y.W.Y. conducted the data visualization and graphical interpretation. L.Y.X. and Y.J.G. provided vital support or finance. All authors made contributions to and approved the final document of the paper before its submission.

## Conflicts of interest disclosure

Conflict of interest statement: The authors declare that the research was conducted in the absence of any commercial or financial relationships that could be construed as a potential conflict of interest.

## Research registration unique identifying number (UIN)

Not applicable. Our article does not deal with humans and is an econometric analysis of the literature within the original database.

## Guarantor

Jiguo Yang.

## Data availability statement

Not applicable.

## Provenance and peer review

Not commissioned, externally peer-reviewed.

## Institutional review board statement

Not applicable.

## Informed consent statement

Not applicable.

## Declaration

I hereby declare that this manuscript has not been published and is not under consideration for publication elsewhere.
